# Tobacco and Education

**Published:** 1897-06

**Authors:** 


### Tobacco and Education.

A crusade against the use of tobacco has recently been started in a number of our American universities. It is a recognized fact that tobacco, when taken into the system in any form, is injurious not only the physical health, but to the intellectual development as well. The results obtained in schools where the use of tobacco has been discarded are very encouraging, and show clearly the harmful effect which this obnoxious weed has upon the system. It is gratifying to note that some of the best colleges of our country have taken a decided stand against its use by their students. The Boston University has issued an ordinance that those students who are unwilling to forego the use of tobacco, while within the precints of the university, will have their fees returned, and their names taken from the books. The Ohio Wesleyan University has made a rule forbidding its students to use tobacco in any form, and other universities have made similar ordinances.

In some of the higher educational institution of this country attempts have been made to obtain statistics as to the effects of tobacco on the academic youth. In 1891 the official physician of Yale published the results of observations on the undergraduates of that university. In a class of 147 students, he found that in four years 77 who did not use tobacco surpassed the 70 who did use it to the extent of 10.4 per cent. in increase of weight, 24 per cent. in increase of height and 26.7 per cent. in increase of chest girth. The most marked difference was, however, in point of lung capacity, the abstainers showing an average gain of 77.5 per cent. more than smokers or chewers. Among the undergraduates at Amherst it was found that during the four years the abstainers from tobacco gained 24 per cent. in weight, 37 per cent. in height, 42 per cent. in chest girth, and 75 per cent. in lung capacity over those who used tobacco.—*Modern Medicine.*

ARTIFICIAL camphor that closely resembles the natural product is now manufactured by passing hydrochloric acid into spirits of turpentine surrounded by a freezing mixture.

